# The Aurora Epidemic

**Published:** 1891-10

**Authors:** E. J. Beall

**Affiliations:** Fort Worth, Texas


DANIEL'S
TEXAS MEDICAL JOURNAL.

A Representative Organ of the Medical Profession, and an Exponent of Rational Medicine; devoted to the Organization, Advancement and Elevation of the Profession in Texas.

PUBLISHED MONTHLY.—SUBSCRIPTION $2.00 A YEAR.

Vol. VII. AUSTIN, OCTOBER, 1891. No. 4.

### Original Articles.

CONTRIBUTED EXCLUSIVELY TO THIS JOURNAL.

*The Articles in this Department are accepted and published with the understanding that we are not responsible for, nor do we indorse the views and opinions of the writers by so doing.*

### THE AURORA EPIDEMIC.

*E. J. BEALL, M. D., FORT WORTH.

"IT BELONGS to a large group of local epidemics of an anomalous kind, the nosological relations of which are not obvious and which can only be understood when they have been carefully studied. Every one of them should be placed upon record with as much fullness of detail as practicable."—*Morgagni.*

Time has calendared the beginning of another year since a local epidemic of infectious disease prevailed at the sister village of Aurora. From a condition of happy home life this pretty and thriving village, nestling in a beautiful valley of the Trinity, was suddenly wrapped in gloom and draped in mourning. Men and women were tested in heroism. The local members of the pro-

*The above abstract of Dr. Beall's paper, and Dr. West's criticisms during the discussion, at Waco, are published herewith as requisite to an understanding of Dr. Beall's reply to said criticism—which follows, addressed to the Editor. Other criticisms he has ignored.—ED.

fession were tried in the crucible of dangerous duty. Their minds were strained, bodies wearied, hearts saddened. Let it be said, however, that Burch, Roark and Bobo proved true to the spirit that has made our calling a handmaid to religion; that their acts were such as always, under every circumstance and in all ages, have characterized the true physician in his rounds of mercy—or servitude, if you wish. The physicians of Aurora, as the writer can attest, proved Mannings indeed and upheld the flag of mercy and encouragement when deserted by nearly all save the sick and dead. At this juncture the writer endeavored to lend, for a couple of days, his feeble aid to the stricken people and wearied physicians of Aurora.

After his return home circumstances forced him to give publicity to certain statements in order to correct wrong impressions that had grown out of messages or letters received from Aurora by the public press of Fort Worth, and in doing so advanced a few thoughts as a mere morsel for the gratification of public curiosity, which at that time was at a high pitch. To these thoughts professional exception was taken, and letters were written urging an attack upon what had been stated. A high sense of propriety possibly prevented those who endeavored to make "cat's paws" of others making the attack themselves. Yet it was threatened in one instance, but so far the writer has not seen the threat carried into execution in the shape of a maiden effort in journal literature.

After the writer's return from the International Medical Congress at Berlin he received the Transactions of the State Medical Association, in which occurs a paper by his friend, Burch, upon the Aurora epidemic. In this paper the doctor criticises very kindly the thoughts that I had expressed, and evidently had in view only the elucidation of certain facts pertaining to the epidemic. However, I must here be allowed to say that in some particulars he did not place the construction intended to be placed upon some points that I made. That the Association may be in a position to more correctly judge the ideas meant to be conveyed, I here present for their consideration a limited abstract.

The first part of the paper, which is the parent of the present, was devoted to the schematic illustration of the points at which the disease at Aurora began and extended until its cessation. For the sake of brevity I have omitted certain portions that are not of special interest or importance. Then follows:

"It is interesting to note that on the 28th of January, 1890, James Jefferson and his mother arrived at Aurora, from the Indian Territory, and stopped at the Jefferson residence, the former sightless and toeless, the result of a disease which had led his mother to go to the Territory for him. The mother had been in Aurora only a few days when she was called to Azle on account of the sickness of another son, William Jefferson. On her way thither she became very ill and was obliged to stop at Dr. Gordon's, near Azle, where she died. It is interesting to note that when Mrs. Jefferson and her son arrived at Aurora the valise of the maimed Jefferson, by mistake, was taken to the Deerberry house, and that it remained there six days. The mistake was not discovered until Mrs. Landers, a guest of the Deerberry house, began preparation for departure to her home near Sunset. Mrs. Landers arrived at Sunset and died two days after leaving Aurora—the first victim of the Deerberry house. Just after Mrs. Landers departed, Miss P. Roberts, an inmate of the Deerberry house, was taken very ill. She was removed to the house of her mother, in the north part of town, and her case proved to be the second victim of the Deerberry house. The Elliott family were at this same house, and on February 8 or 10 the two sons, Cam and Zeb, were taken sick. They weie removed two miles into the country, where their mother was also stricken shortly afterwards. Opposite the Deerberry house, across the street, lives Dr. Burch. Two of his sons were seized within an hour.

"It was stated to me that after the arrival of Jefferson and his mother, the bed clothing upon which his wife and child died in the Territory, were hung upon a line at the corner of the residence; that it was the custom of Clements and Watkins, who were seized with the disease on the 10th or 12th of February, and died at the southwest corner of the square within one hundred feet of each other, to visit daily the stable situated in front of the residence. Forty or fifty feet west of the store in which Watkins was seized lives Mrs. Covey, whose daughter had been seized, and whose mother, taken subsequently to the daughter, died within twenty hours from the onset of the disease. Young Deerberry, whose place of business was near the southwest corner of the square, was an early victim, perhaps the first; he slept at the Deerberry house.

"The physicians are uncertain about this last case, so I exclude it. There is also an uncertainty in regard to Mr. Freylan, who had slight illness about the same time that Mr. Deerberry was sick. He was seen at church and afterward seated upon some lumber near the clothes line upon which Jefferson's bed clothing was still floating with the breeze, on the Sunday previous to the Friday on which he was seized.

"Now, if information (which I am at present seeking for other purposes than the secular press) shall show that James Jefferson, his wife and child, died of the same disease which has lately afflicted Aurora, the inference will be too plain to admit of cavil that the disease is portable, that it was introduced into Aurora by the valise, person or clothing of the parties named. This will be further strengthened when positive information concerning the particulars of the sickness and death of Mrs. Jefferson, near Azle, and Mrs. Landers, near Sunset, is received.

"What is the disease at Aurora? This question the laity have asked to gratify curiosity or to back crude opinion upon equally as crude knowledge. It is a question of interest to the physician, that he may direct a line of thought in a correct channel, that he may more intelligently adapt therapeutics to pathology.

"Is it typhus fever? While one or two of the symptoms observed are common to that disease, too many of its symptoms are absent; besides, we know that true typhus is rare in this age, in both the old and new world, and when introduced into this country from foreign lands, as occurs now and then, its contagious and fatal nature is so well recognized that prophylactic medicine rears a shield of defense against it, so that the disease is strangulated as soon as it is transferred from the merchant ship to the shore, thus preventing its acquisition of a foothold in our maratime ports.

"Is it cerebro-spinal fever? If it is, four limited epidemics participated in by the writer, were not. Thirty years observation in this latitude, and frequent and close observation at the largest medical centres, has not furnished him with an opportunity of seeing the disease he saw and studied for two nights and days in our neighboring county. In cerebro-spinal fever the delirium is active and the mind is perturbed. In the disease at Aurora, apart from the slight delirium of the incipient fever, the mind is clear and collected. I saw no manifestatation of cerebral disturbance of an organic nature. The Elliotts were mentally undisturbed; Miss Covey's mind was clear as a bell—her intelligence and mental calmness under the severe pain, mental and physical anguish, only presented the index of the sweet disposition which has won for her among her friends the reputation of being one of the loveliest of her sex; little Mack Burch had no delirium; his mind was clear and calm; same condition existed with Miss Peach Roberts. When sudden apnoea (respiratory failure) was pending in the case of Charlie Burch, then carbonized blood obtunded his bright, bright mind—not disease within the cerebrum, membranous or otherwise. There was no rigidity of the cervical muscles in other cases than the one mentioned, and that was due to a lesion of the spinal cord or toxic effect upon the respiratory center, and this was the lesion or impression that induced the apnoea which extinguished his life. In some cases there was a mulberry rash, size of spots varying from that of a pinhead to a silver twenty-five cent piece. This eruption is seen in cerebro-spinal fever; but oftener there is a vesicular herpetic or erythematous eruption occurring later in the disease than it did in the Aurora cases; this eruption I did not see in a single instance. Several of the cases at Aurora presented upon the skin large dark bullæ (rupial) filled with *bloody serum*, when broken and collapsed, *resulted in sunken*, *dark brown*, *smooth scabs*. The bullæ seemed to show a predilection for the knuckles, the joints of the toes and over the carpal and tarsal bones. This eruption I had never seen in cerebro-spinal fever.

"The most important symptoms observed in the disease at Aurora were the arthritic (joint) disease—peri and intra-articular. This was the prominent symptom. I have not seen nor have I ever read that this lesion was ever connected with *cerebro-spinal fever of late years.* In the knee joints of two cases I could discover *large effusions of serum*, *or blood and serum.* In cerebro-spinal fever it is not uncommon for the eyes to be destroyed by suppurative choroiditis. In two of the Aurora cases, Miss R. and one of the Elliotts, blindness existed, the former having lost one eye, the latter both. Jefferson had, in addition to bilateral blindness, joint disease which resulted in a *large slough upon the dorsum of the foot and the loss of several toes.* In cerebro-spinal fever, as well as in the Aurora fever, hyperæsthesia (great sensitiveness) of the skin existed. I regret that the insufficient light and absence of proper instruments prevented an examination, which would have determined whether the eye lesions presented any difference from those belonging to cerebro-spinal fever, as generally seen.

"The fever at Aurora and cerebro-spinal fever *certainly present symptoms common to each other;* but there are other symptoms so entirely different that, to my mind, the fever at Aurora is entitled to separate study. Both diseases surely belong to the acute, infectious maladies, and are, to a limited degree, contagious. They may prevail (rarely, however,) as a sporadic disease, but generally as an epidemic, though usually limited as to extent of country invaded, and duration of existence in a given locality. If cerebro-spinal fever is contagious, as stated by Professor J. Lewis Smith, in his classical article upon that subject in the recent work of Keating, and, as Professor Stillé also says, though not strongly contagious, we must claim a like limited contagion for a disease so nearly allied to that disease as the one which, apparently, is now waning in an adjoining county. We may infer that the disease in question is of microbic origin. While its germ has not yet been isolated, or experimentally cultivated, reasoning by analogy, we may conclude that its nature is such.

"Those diseases which science has proved to be due to micro-organisms have certain concomitants, curves, crises, etc., thereby enabling us to satisfactorily determine that a like nature exists in regard to a given disease, although its *materies morbi* has not been demonstrated. The comma bacillus of cholera is now well known; the rod shaped bacteria of Klebs is recognized whenever the etiology of typhoid fever is under consideration; and that of Friedlander, the micrococcus of croupous pneumonia. These we know are essential diseases; belong to the acute infectious class; may become epidemic. By the history, nature and analogy manifested by these diseases, we may conclude that the disease in question is of a like class and has certain attributes in common—infectiousness or contagiousness and epidemical disposition. If it is true that a micrococcus, like that of pneumonia, has been determined as belonging to cerebro-spinal fever, this disease and the Aurora fever being similar in some features, though dissimilar in others, we may infer an electiveness that will confer contagiousness and epidemicity upon one as well as upon the other.

"This disease, whatever its undetermined true nature may be, while not so heart-rending as the usual visitations of cerebro-spinal fever, because of the absence of serious contortions and delirium, in results, perhaps, should be more dreaded. Six out of twelve dead, and three out of the six living were better if dead. What a picture!

"I think the nature of the disease at Aurora is a hæmaclytic fever with peri and intra-articular inflammation, with effusion of bloody serum into joints, and late in the fatal cases into brain cavities, hemorrhage into the skin, with tendency to sphacelation, etc.

"I do not believe the same disease has existed upon the American continent within seventy-five years. Jackson and others, between the years 1806 and 1815, described the disease under the term 'spotted fever.' A number of papers upon the disease were read and discused, referring to it as it prevailed in Massachusetts, within the dates mentioned.

"In no case that I have seen of cerebro-spinal fever, nor of which I have read, were the prominent symptoms exhibited that I saw at Aurora, except those referred to in the foregoing."

Should the writer revise the foregoing he probably would modify a portion thereof, but in the main he has no retraction to make. What he wrote was in the interest of free, independent thought, and is in keeping with the means by which medical progress has been advanced in every age of the world.

By way of illustrating that our ideas sometimes undergo change, an incident of many years ago comes to mind: The writer had contended for the essentiality of croupous pneumonia from his earliest entrance into medicine, and, many years ago, wrote a paper upon the subject, which was published in one of the earlier numbers of a small journal edited by Dr. Greenville Dowell, of Galveston. Just before publishing this paper he incorporated the main ideas thereof in two letters, one of which was sent to the late Dr. Austin Flint, and the other to Dr. Alfred L. Loomis, for the purpose of eliciting an opinion thereon. Dr. Loomis replied that the idea was preposterous. He now thinks and teaches very differently. Professor Flint replied that for several years he had entertained such views in a crude state. Ere the year closed he read a paper upon the subject before the State Medical Society of New York, and ever afterwards taught the doctrine in Bellevue.

I am aware that the books teach that cerebro-spinal fever, cerebro-spinal meningitis, or spotted fever, presents different symptoms in different cases and between cases in the same epidemic. So often is this the case that Stillé has written of cerebro-spinal fever as the "chameleon-like" disease.

In the paper herein mentioned I say that the disease at Aurora had many symptoms which belong to cerebro-spinal fever, also symptoms which in some respects resemble typhus fever; but I did not make a diagnosis—a statement of the nature of the disease was not intended for its nomenclature. Hæmaclytic (a word I coined from the Greek) spinal fever referred to the blood disintegration. Is there authority for the ideas of the last two paragraphs? Verily, it will be found in the nomenclature of writers as varied as the symptoms mentioned by them. Examine Wilson and see the synonyms he records under the title of cerebro-spinal-meningitis. These synonyms are measurably based upon the different views of the nature of the disease or diseases in question.

Referring to the text at the beginning of this paper, which emanated from one of the ablest men of our profession in America, who received the article upon which Dr. Burch predicated his paper, and from whose letter I made that abstract, the ground I endeavor to maintain is surely tenable. Who will contend that there do not exist substantive diseases that are described collectively under a general title as cerebro-spinal fever? To say to the contrary is to imply that medicine has reached its ultimate development, and to set at naught that which we know to be tiue. How long has it been since typhoid fever, a substantive disease, was included with typhus? How long since measles and scarlet fever, substantive diseases, as we think, were included under the term measles? In this way, by scanning the nosology of a half decade, much less one, two or three centuries, I could almost interminably demonstrate the position taken. To ignore this idea is to put a brake upon the wheels of medical progress; to stay its advancement; to measurably negative that which improved work with improved diagnostic aids may do; put an end to the accumulated advanced thought of the present, and relegate progress to the years and ages calendared in the decades and centuries of the past. In the absence of definite knowledge of the etiology of the disease and the essential nature of the infective principle, is it not true, perhaps, that we cannot at present say whether cerebro-spinal fever, so called, with its diversity and irregularity of symptoms, is one substantive disease, or several substantive diseases included in one term? To the writer, there are pertinent reasons for thinking that the numerous local epidemics of disease included under the term cerebro-spinal fever are in many instances diverse in symptoms and nature. And just here in the field of medicine is the arena for original work and thought.

Reasoning from analogy, may we not conclude that in all probability in many instances we include several substantive diseases in a common term? Not only is this true in regard to the disease in question, but also in other diseases. Are not many of us becoming disgusted at the wide range given to the term malaria? Who of you but believe that this term is too often resorted to as a cloak for individual or professional ignorance? The writer has passed through several local and general epidemics of disease nosologically related to cerebro-spinal or spotted fever; but at Aurora he saw cases whose history and semeiology differed from what he had seen in previous visitations. Take up any ordinary work on the practice of medicine, read the article on cerebro-spinal fever, and observe how many symptoms mentioned by Dr. Burch and myself are conspicuously absent from the description of the disease. If Dr. Burch will recall satisfactorily to his mind the cases he referred to which he saw in Kentucky and Mississippi, I doubt whether he will be satisfied in memory that these cases showed the same comparative absence of *cerebro-spinal lesions* and the presence of the same *skin* and *joint lesions* that the Aurora cases did. Take Watson, Roberts, Niemeyer, the able monograph of Deering Roberts, Barlow, and many others to whose writings I might refer, and you find that many of the prominent symptoms seen at Aurora are not mentioned. I am aware that Wilson and Stillé in their classical and exhaustive articles upon cerebro-spinal fever mention many of the prominent symptoms seen at Aurora; *but did they ever see cases with these symptoms?* I do not believe they did. I believe that in their efforts to exhaust the subject the Boylston Library, at Boston, has been drawn upon; and that they present not what has occurred in their personal experience only, but, medical historians as they are, have brought down to the day of their writing the features which belong to a disease as it appeared in the younger decades of the century, and which living writers have not seen.

My observations at Aurora recalled the remark of Ziemssen, that "it is difficult to understand why the American physicians continue to call cerebro-spinal fever, spotted fever." It is because one writer, copying after another, speaks of spots which were peculiar to the epidemics of 1806 to 1820, and to which I think the Aurora fever was allied or more nearly resembled. This eruption was peculiar to every case I saw at Aurora. It was peculiar to the case I saw in Fort Worth before the outbreak at Aurora, and to the case I treated in connection with Dr. Van Zandt at the Mansion hotel after the Aurora epidemic, which last case was in the person of a resident of Quanah, who was taken with a chill on the cars near Hodge, during a business trip to

Fort Worth. I believe that the occasional cases (many of which doubtless we have no knowledge) that have occurred along the tributaries of the Trinity river, and perhaps the cases now appearing at Mesquite, will all indicate the peculiar eruption I mentioned as occurring at Aurora, which, as stated, was purpuric in character and not vesicular, as usually obtains; the former showing blood or capillary involvement, the latter an undefined neurotic condition. This great dissimilarity of skin and joint manifestations is sufficient to interest the attention of the clinician in establishing his semeiology, and the pathologist in recording the lesions found on post mortem.

In the epidemic of the disease generally described the eruption is vesicular. "Herpes is the most common, and is usuallly confined to the face, but may appear upon the trunk, as herpes zoster, or in circumscribed patches upon the limbs." In not a single case at Aurora did I see this. In most of the cases hitherto seen by the writer, in but few, if any, was there joint disease with effusion, swelling, pain, which was the *most distressing symptom* observed at Aurora. In prior observations he had not *seen bullæ filled with bloody serum, rupturing and drying into excavations with dark brown scales, in two or three instances about the toe joints, causing them to fall off.*

The screaming delirium, with the head drawn to the heels, eyes red and distended, I did not see at Aurora. Occasional pain was complained of, but the minds were generally clear, the joints were generally involved, and to the joints one's attention was directed by the sufferer in advance of every other symptom or condition. Another feature differing somewhat from prior observations refers to the ages of those seized. Twenty-five per cent of the cases were 60 or more years of age; fifty per cent more than 25 years of age. This fact is perhaps without precedent in former local or general epidemics; children being more liable to the disease than adults.

*I do not wish it understood that I did or do state that the disease* (to which I have given but little thought during the past year, and about which I am now, just before the Waco meeting, devoting only a night to, without reference to authorities), *is not cerebro-spinal fever.* I did state that in many features the epidemic resembled cerebro-spinal fever or cerebro-spinal meningitis; in some features typhus fever. This is not a strained comparison; for proof look at the synonyms that have been given of the disease: Cerebro-spinal fever, cerebro-spinal meningitis, typhus fever, petechial fever, malignant purpuric fever, pestilential purpura, febris nigra, etc. These various synonyms indicate to the writer's mind that the nosological relations of local epidemics occurring now and then are anomalous in character, and point to grave uncertainty of their true nature.

By way of further illustration of this point, Leyden states that among the German troops before Paris in 1870, a series of cases occurred in which marked rigidity of the neck, severe headache and hyperæsthesia were present, while the abdomen was flat, the temperature was low, and the bowels were confined. It was thought that these cases were cerebro-spinal fever; but the crucial test, an autopsy, proved them to be cases of typhoid fever, but with slight implication of the bowels. Aitken, I think, refers to similar cases. I have seen six or eight cases, such as Leyden and Aitken describe, in the city of Fort Worth. The patients complained of stiff and painful necks, a painful arm or leg, headache and intense hyperæsthesia.

Surgeon Jackson, U. S. A., in his report of 1861, says that among the troops under his charge, near Annapolis, Md., there occurred a number of cases reported by some surgeons as typhus fever, and by others as spotted fever. Jackson denominated the cases malignant congestive fever. There were pethecial spots (hæmaclises—disintegrated blood), and the blood did not coagulate in fatal cases. The absence of the usual brain symptoms during life was opposed to cerebro-spinal fever. The mind in many of the fatal cases was clear to the last. The autopsy showed the cases to have congestion of the brain membranes, without deposits of lymph or pus.

Hirsh, of Erlangen, in his work upon cerebro-spinal meningitis, is unwilling to concede that the spotted fever which prevailed *in the United States from 1806 to 1820 was cerebro-spinal meningitis.* This opinion he emphasizes in his great work published at Berlin in 1866. Radcliff, in the article on the disease, in Reynolds' System of Medicine, maintains the same view.

That we have much before us, quite a field of work, when we take into consideration the irregular and varied symptoms grouping in various outbreaks of the infectious class of diseases, there can be no question. Does climate do this? Do the habits of peoples bring this about? Or is it more probable that variations of types are rather the manifestation of the reaction of different organisms to a common infection?

Again, we pretty generally admit at the present time that the infectious class of diseases is due to certain micro-organisms. In some infectious diseases the micro-organisms have been satisfactorily determined—in others nct. So sure are we of this fact as regards some diseases, that we safely infer, though as yet ignorant of the special *materies morbi*, that the near future will determine the infective cause in all this class of diseases. Admitting this, diversity of symptoms must be accounted for on other grounds. Suppose we call to our aid the science of bacteriology. We then enter a field in which order is found—is paramount. No irregularity must exist here, it is contrariwise to nature; irregularity in symptom grouping must give way to regularity. Symptoms described and applied collectively must undergo division, separation, and substantive diseases find their proper place in our nosology. We are constantly doing this, and have done so since progress marked the science of medicine. In the etiology of disease traced to microscopic life there is an order that marks the higher orders of creation. If a causative factor of disease is found here, the effects must semeiologically and pathologically show order. We can not find scabies without itch—nor will a tinea be found with itch; otherwise irregularity and disorder would be seen. There would be collective symptomatology instead of substantive symptomatology.

These beliefs lead me to predict that in the future some of our profession will bring order out of disorder, and that finger-boards will be erected by which diseases now confounded together will be separated, and their proper place designated in the category of diseases.

Want of leisure has prevented me rewriting this hastily gotten up paper. I do not especially refer to the paper of my friend Dr. Burch; for I only hurriedly scanned it over some days ago. What I have written is very inadequate to my wishes; but I have no time to cut out, rearrange, or give the views of others in corroboration of my own. And I close, indulging the hope that whenever the infectious epidemical diseases raise their standard, and the arrows of death are taken from the quiver, that we will remember that a close study of disease and proper symptom grouping will enable us to better serve our kind if we shall winnow substantive from collective diseases, for many outbreaks of such diseases are anomalous in kind and their nosological relations in the present state of science are illy understood.

DISCUSSION—(NEXT DAY).

DR. H. A. WEST, of Galveston: Before the minutes of yesterday are adopted I wish to make a few remarks on the paper of Dr. Beall. As I occupied the chair at the time, I was prevented from participating in the discussion which ensued after the paper was read. I am sorry the doctor is absent, as I wonld much prefer that he was present to reply and explain his position more fully. The discussion of yesterday mainly concerned the publication of the paper, leaving the real merits of the question unanswered. As there are some things in the paper I do not wish to leave untouched, I trust you will bear with me a few moments while I call them to your attention.

Dr. Beall, in my opinion, has made several mistakes in regard to the subject under discussion, and his position now, as shown by the paper presented yesterday, illustrates the difficulty a man has in getting right after he has once gotten wrong. His first mistake was one of diagnosis, for whatever his opinion now as to the nature of the disease at Aurora he evidently differed from Dr. Burch and others at the time. If he agreed with those gentlemen that the disease was epidemic cerebro-spinal meningitis, what was the necessity of Dr. Burch's paper last year at Fort Worth? But we have Dr. Beall's own words upon this point. He asks: "Is it cerebro-spinal fever?" And replies: "If it is, *four limited epidemics participated in by the writer were not.*" "Thirty years observation in this latitude, and frequent and close observation at the largest medical centers, has not furnished him with an opportunity of seeing the disease he saw and studied for two days and nights in our neighboring county."

He constantly speaks of the disease as "Aurora fever," and says: "I think the nature of the disease at Aurora is a hæmaclytic spinal fever, with peri and intra-articular inflammation, with effusion, hemorrhage into the skin," etc.

But he says: "I did not make a diagnosis. A statement of the nature of the disease was not intended for its nomenclature."

Well now, if Dr. Beall is not trying to prove that the disease at Aurora was something else besides cerebro-spinal fever, what is all this talk about it? Evidently he made a mistake in diagnosis. Dr. Burch has proven that. What is the next mistake? In attaching so much importance to the *symptoms*, and apparently ignoring entirely the pathology of the disease, and the fact that the symptoms, multiform and numerous as they are, can be accounted for by the nature, extent and anatomical location of the lesions.

With this fundamental fact kept in view, it is easy to understand why the symptoms may vary so much in different epidemics, or even in the same epidemic. In one instance there may be lepto-meningitis chiefly, which may predominate in the pia mater of the cord or the brain. In other cases the inflammation may reach the underlying parenchyma. In others there may be numerous centers of genuine encephalitis or even abscesses of considerable size. With such a variety of lesions affecting the central nervous system, is it any wonder that the clinical picture may be almost infinitely modified. Dr. Beall lays much stress upon the absence of cerebral disturbance, delirium, etc. What easier explanation is wanted of the fact than that the disease was located mainly in the cord? But is that any reason to cast a doubt upon the diagnosis of cerebro-spinal fever? I think not.

But what is the doctor's next mistake? In my humble judgment he made that when he went to his dictionary to conjure up a new name for a disease that now has already too many names; in fact, he has given us two new names, "Hæmaclytic fever" and "Aurora fever." I wish to enter an emphatic protest against such nomenclature. If he keeps on at this rate he will have a new disease for every cross-road and blacksmith shop in the State.

Now for mistake No. 4: If the doctor was in doubt as to the nature of the disease, why did he not make an autopsy and settle the matter once for all? "More investigation and less discussion" would apply in this case as well as to another subject mentioned in my report.

Let me mention one other point. Dr. Beall refers to the arthritic symptoms as something phenomenal, and as indicative of some other disease. If he will look at his books again I am sure he will find the joint trouble described as frequently occurring in epidemic cerebro-spinal meningitis, which to my mind was unquestionably the disease prevalent "in our sister village Aurora."

---

### REPLY TO DR. WEST'S CRITICISM.

Editor Daniel's Texas Medical Journal :

An important land suit called me to Throckmorton at the time of the meeting of the State Medical Association at Fort Worth ; several cases of abdominal surgery prevented my attending the meeting at Waco. I forwarded a paper to the latter place, or rather, sent an infernal machine, which my friend, the gallant Raines, exploded in camp. The article abstracted as briefly as my leisure will permit, I enclose, and ask its publication, together with the criticism of my friend West, of Galveston.

There were other criticisms that need not be referred to, as they indicate too much ignorance or malice—and to the writer's mind reflect very seriously upon Dr. West and the publication committee. On West, that he should condescend to reply to "so much of nothing"—on the publication committee, that they should condescend to prostitute the transactions to worthless purposes. I will here state, by way of parenthesis, that the paper that contains "less of something*" bears the same ear-marks as the report of the Chairman of the Section on Surgery of the Texas State Medical Association, of 1885, which report was characterized by several journalists as being among the best reports that emanated from any State medical association during that year.

The paper read at Waco was hurriedly written—was intended more especially as suggestive, and to direct a line of thought in certain channels of interest and importance,—was not "toned down," had less oil on the manuscript than that of the critics, and emanated from a busy doctor, who neither lectures nor preaches, and, perhaps fortunately, writes but little.

But to the paper and the criticisms, and the effort to sustain some of the points presented, if there were any, and a weak defense of some of the assertions to which exceptions were taken; I still repeat that I did not make a diagnosis of the disease at Aurora. The nature of a disease is by all systematic writers considered disconnectedly from the nomenclature, diagnosis, prognosis, etc. If I had in view the direction of attention to unusual and anomalous symptoms, would it not be legitimate for me to describe such as had passed under my personal observation, to the end that others more competent and with better facilities might seize the torch, like the Romans of old, and bear it onward till co-operative thought and study had the plainer made a suggestion—that hypothesis might be merged into fact, and more satisfactory conclusions reached?

During thirty years of active practice I have participated in several epidemics of cerebro-spinal fever. I have never doubted having seen the disease. There existed no difference of opinion between myself and colleagues as to the cases or epidemics in

*Dr. H. W. Dudley's criticism.—Ed.

which we labored together. But in the disease under consideration I observed peculiarities that I had never before seen — *that I believe living writers and observers in America had never seen.* If now I had a reasonable hope that I could substantiate this proposition, which I can do, is it not legitimate and proper that I should do so? Away with a philosophy or logic that would teach contrariwise!

Just here is where "so much talk comes in;" and upon this I predicated the hope or belief that some medical Columbus would be furnished by a progressive future, who would erect finger-boards by which diseases now confounded together would be separated,—order brought out of disorder.

Will the gentlemen who are so sure of the nature of the disease, the mention of which created such a stir at Waco, and who are so well satisfied with its nomenclature, and who insisted so strongly upon my mistakes, allow me to ask if *they* ever saw the skin and joint manifestations I detailed? or can they point me to a writer who ever did, who does not refer to the Irish epidemic of '66, or the Massachusetts epidemics of the earlier part of the century? If they cannot do so, and I have no fear of their doing so, I pray, as Dr. Pepper wrote me, "for a letter of particulars," and urge them when the opportunity presents, to carefully group symptoms and spare not the scalpel, and endeavor to establish a proper nosological relation for such diseases.

The gentleman refers to the "absence of cerebral disturbances, delirium, etc., and said that such conditions could be accounted for upon the hypothesis that the force of the disease was expended upon the cord. If this were true, why were other symptoms of cord lesion absent, and which of necessity must have existed if the lesion was so located? Did he ever see an epidemic of spinal meningitis?

The gentleman's strictures with regard to the term "Aurora fever," are quite puerile. Without a term of designation as to locality it might have been inferred that "Roman fever," "Chagras fever," "African fever," or the "modified cerebro-spinal fever" of Williamson county was meant. A stretch of imagination only could cause one to think a reflection was intended upon the village of Aurora, or that the term was intended as a permanent name for a disease that rarely prevails, if it be ordinary cerebro-spinal fever, or likely to do so if akin in nature, only as a disease to be dreaded, and "if modified" the modification certainly has no reference to the death rate of which I have any knowledge in medical history.

If I conform to correct rules in word making—keep within the languages from which our language is derived, and a place well supplied the term, euphonious and expressive, why may not he or I coin a word for adoption? The language he and I use was so made—is it yet perfect?

The prejudice, away from large cities, with regard to autopsies is a great drawback to the country practitioner. We do not have the opportunities for knowing how often we make mistakes compared with our city cousins. Rural predjudices doubtless shroud many an important fact within the breast of the country practitioner, and unquestionably retard pathological work.

Referring to the last paragraph in friend West's criticisms, it is passing strange that after 24 hours study he should refer me back to my books, that I may learn that arthritic symptoms belong to the category of cerebro-spinal fever symptoms. This indicates that had he given a liitle more attention to the object of my paper, and less to a disposition to criticise that which was never intended by me, his remarks would have fallen into a different rut. While I know arthritic symptoms occur in cerebrospinal fever, many epidemics do not present such symptoms. I can indicate several interesting monographs upon the subject in which such symptoms are not mentioned. I saw such symptoms in the epidemic of '63. But will he *find the observer or the writer who ever saw the joint symptoms I detailed as occurring at Aurora?* if he will except the Irish epidemic of 1866 (see Reynolds), and the spotted fever of the early part of the century, in New York, Pennsylvania and Massachusetts? When he has found the writer, or personally meets the observer who has shown, and can demonstrate that he has not drawn upon the literature of the Irish or the New England epidemics, let me repeat, as Dr. Pepper wrote, "Pray write me."

The above is written to elucidate the preceding abstract of the Waco paper, as well as to answer some of the criticisms therefrom. As my ideas are presented in a different suit of clothes, probably they will be better understood and the reader will conclude that really after all, there was "much ado about nothing."

In the elucidation of a proposition it is well sometimes to sustain it by corroboration of the opinion of others. When this is done, the standing of those upon whom we rely will add to, or detract from the force of the proposition presented. With this in mind, I present a circular letter which I addressed to several medical acquaintances, and append the replies received :

"FORT WORTH, TEXAS, August 12, 1891.

"DEAR DOCTOR :

"In your observation of the arthritic symptoms occurring in cerebro-spinal meningitis have you ever seen the synovial sack filled to an extreme degree with fluid—perhaps bloody serum ?

"In your observations of the skin manifestations of the same disease, have you at any time noticed disseminated, dark purplish spots over the body, and upon the feet and hands bullæ as large as the end of a finger, filled with bloody serum, which upon being ruptured, leave an excavation in which soon forms a dark brown scab, and in some cases, where over the toe joints, were followed by a destructive process to an extent that toes dropped off ?

"Can you cite me to any writer who has ever reported such ?

"Yours truly, E. J. BEALL."

To the above letter the Nestor of American Medicine replied :

"*Dear Dr. Beall—I have not seen any cases presenting the symptoms and changes you describe. Yours truly,*

"N. S. DAVIS.

"CHICAGO, August 14, '91."

It is unnecessary for me to direct attention to the writer of the above letter, the father of the American Medical Association, an ex-president of the International Medical Congress, a practitioner and lecturer of more than a half century, the author of one of the most complete and popular works on theory and practice, in fine, the Nestor of American medicine.

Prof. D. J. Roberts, the author of an elaborate paper upon cerebro-spinal fever, writes :

"DEAR DOCTOR—Yours of the 12th inst. received. In reply to both interrogations, *I answer in the negative*. Although I have seen cases of cerebro-spinal meningitis, I cannot regard my personal experience as very extensive, *and have seen none of the pathological conditions mentioned*."

Prof. Shaw writes that cases with purplish spots, vibices, etc., are noted by Reynolds, and refers me to that author. I am familiar with Reynolds. What he writes is predicated upon the epidemic in Ireland in 1866, and which, I am satisfied, is allied to the epidemic of the first quarter of this century in the United

States, and as I claim, with the disease of our section and now being considered.

Dr. Pepper, whom I regard as the Leyden, the Trousseau of American medicine, replied that "*such manifestations must be very rare*," and urged that I give him particulars in regard to the cases.

The above extracts show very plainly that the joint symptoms and skin lesions seen at Aurora have not occurred under the observation of a few of the acknowleged able men of our profession in this country.

I now propose to present an abstract of the symptomatology and pathology of the epidemics of the N. E. States as embodied in a report by a committee of the Massachusetts Medical Society in 1810. If this report sustains the position taken, and I furnish the best of authority that the disease which the abstract refers to was not cerebro-spinal fever, then the Aurora epidemic was not, my object is achieved, and further discussion is estopped. I regret that JOURNAL space interdicts presenting this interesting abstract in its entirety. It is well to bear in mind that upon the committee appointed by the Massachusetts Medical Society to collate and report upon the spotted fever of New England, in June, 1810, were men—intellectual giants—who have left their impress upon the medical literature of that age and section, and whose descendants yet shed a halo of glory upon the old Bay State ; for the Jacksons and Warrens still live, as they did in the early times.

But to the abstract : "The invasion of the disease is sudden and violent, shifting pains in one joint or one limb, often in a finger or toe, in the side, stomach, back, neck or head. Sometimes sensation is like the stinging of a bee, frequently is a most excruciating pain. The pain often moves towards the head and often to one side of the body, more often the left. The pain in the head is compared to the beating of hammers, and the patient thinks he will become crazy. Partial loss of sensibility and paralysis are sometimes first symptoms. The power of sight is affected variously. The sensibility of the skin and parts subjacent is diminished, and the limb feels as if it had been asleep. Sometimes the disease begins with delirium, sometimes with mild stupor and coma ; likewise convulsions and spasms follow in the later stages of the disease. In whatever form the disease commences, there is sudden loss of strength, chilliness, dry skin pale and mottled, eyes glassy, nose contracted, the face sublivid with paleness around the mouth. The whole body becomes cold, respiration laborious, pulse feeble, depression of spirits ; the patient apprehends death, complains of faintness ; precordial distress, fullness of stomach and vomiting. These symptoms become modified in 8 to 24 hours. Within that period some have died, but usually symptoms of a second stage supervene. The pulse improves in fullness and regularity, skin warm, countenance flushed, repiration more regular, eyelids swollen, with pain in the head.

"Among females the following is given as occurring in some cases: Universal deadly coldness--skin white and smooth as marble—coutenance placid—no distorted muscle—pulse at the wrist imperceptible—heart beat scarcely felt—respiration feeble and gasping;—but even from this deadly state patients recover.

"In many cases the skin is remarkably dry at first, then sweats occur upon the face, head and upper extremities, with an offensive odor like that arising from a dead rat in the wainscote of a room. In many cases the skin is remarkably smooth, but not always. The spots on the skin are of various descriptions, occurring more frequently after the first day. Sometimes a rash or miliary eruption only occurs, or a few blotches on the inside of the elbow and other similar parts. The blotches are red and fiery. An appearance like measles has been noted; also pustules like vaccine and variolous eruptions. These are torn by scratching, *after which or without being torn are followed by scabs of a brown color.* These affections are attended by itching most severe on the third day. Oftentimes this is extremely violent, but occurs with the spots when the more important symptoms begin to abate. In some cases *vesicles like blood-blisters*, the size of a large pea, have appeared. In a few days these broke, *discharged blood and scabbed over.* Cases occurred in which *blisters resembling those produced by cantharides* occurred on second and third day on breast and foot. *In one case five inches long and one broad. On the fourth day they became black and dry and the skin sphacelated.* The appearance of petechiæ and vibices was common to the severer cases and of more portent the darker the hue. This did not always appear in fatal cases, nor were they confined to such.

"It is not easy to determine in how large a proportion of subjects the skin is affected with spots and eruptions. One man stated that in eighty cases, of which twenty were severe, he had seen only four in which spots and eruptions had occurred, and these were not among the worst cases. Another estimated the proportion to be two-thirds of the whole. Desquamation of the cuticle and more rarely œdematous swellings of the extremities have occurred at the end of the disease. Glandular swellings are very rare, but swellings occur sometimes in the joints and limbs. These are sore to the touch and look like gout, and the parts affected feel as if they had been bruised. These swellings arise on smaller as well as larger joints and are often purple. This inflammation is called by some erysipelatous; soon after the patient expires, and sometimes before, the skin assumes a formidable livid color."

It would be indeed interesting to the student to present more of this report of the Massachusetts Medical Society, of 1810, but I know JOURNAL space will forbid. The pathology as presented I must briefly refer to as being at variance with the lymph and pus formations usually found connected with the membranes of brains and cord in cerebro-spinal fever. The heart membranes were found inflamed—arterial coats inflamed—also inflammation within the cranium. After death, when the cranium is separated from the dura mater, this membrane usually discharges a great quantity of serous fluid. This is not always transparent like water, but sometimes quite red. The longitudinal sinus is filled with blood and when wounded, discharged great quantities, which pour into it from the central veins.

It is stated in this report (of 1810) that such cases as had the vesicles, bullæ, blood blisters, and which, when torn collapsed and scabbed, were likely to recover, or in the words of the report: "That the eruption, when perfect, gives relief, and that in consequence of it alone, without any other sensible excretion, the disease was entirely resolved."

Now, at Aurora this was the case. The first case (young Jefferson) in which spacellation destroyed the toes,—and this sphacelation was consequent upon dark bullæ or "blood blisters" and destructive inflammation within joints—recovery ensued. In cases of father, mother and son, removed from the Deerbury House to edge of village (Doctor Bobo's cases), recovery ensued in each. In each were the dark spots—petichiæ, vibices, bullæ, dark, and filled with bloody serum; joints filled with bloody serum; sphacelation two inches or more in extent on dorsum of foot, in the case of the younger man.

I forwarded the Massachusetts Medical Society Abstract, secured through a friend in Boston, to Dr. Calaghan, of Mesquite.

The Doctor replied: "I read with great interest this account of a disease so similar, in many respects, to the epidemic at this place, and being written so many years ago, is doubly interesting to me."

I shall forward the abstract to-morrow to Dr. Paschall, of Mesquite, and will look anxiously for his comparison of the cases of the disease at M. and those referred to in the manuscript.

I stated in original paper that the author of the article on cerebro-spinal fever in Reynolds, thought it strange that American physicians still refer to the Massachusetts, New York, and Pennsylvania epidemics as cerebro-spinal meningitis. I here append statement from Hirsch's work :

"Professor August Hirsch in his Handbuch der Historisch-Geographischen Pathologie, Erlangen, 1860, Vol. 1, page 165, refers to the sinking or spotted fevers of earlier American authors, and expresses his disbelief that they may be the same as cerebro-spinal meningitis. In his 'Die Meningitis Cerebro-Spinal Epidemica,' Berlin, 1866, he says, that an epidemic form of disease known as 'typhus syncopalis,' or 'spotted fever,' or 'sinking typhus,' was reported in 1806 to 1822 in many regions of New England and in New York and Pennsylvania, and merely refers to the statement made by him in the Handbook, as above quoted, that he does not believe this to be the true cerebro-spinal meningitis."

This will end what I have to say with regard to the, at least unusual disease I had occasion to see with my friend Dr. Burch, of Aurora, which led to my short, unsatisfactory paper, read at Waco, and which was very harshly criticised. The animus of this criticism is well understood, at least, in North Texas.

I must bear the criticism of tautology, when I repeat that my paper was simply a suggestive one—was intended to direct attention to the more close observation of disease. I have nothing, in any particular, to retract of what I have said. If I have not established my case, the jury, the profession of Texas, can say so. I perhaps can survive the verdict, and if convicted, I shall suffer the punishment; but will hold aloft a flag bearing the motto : "Perfection in the knowledge of disease is yet in the future." Let us not stifle honest thought and conviction by arrogant, unsupported assertion. E. J. BEALL, M. D.

FORT WORTH, TEXAS, Oct. 9th, 1891.

