# Erratum

**Published:** 1891-10

**Authors:** 


ERRATUM.—Omit *Morgagni* at beginning of Beall's paper. Name belonged to a Latin quotation in original, stricken out in copy sent us. This quotation is from a letter from Wilson, of Philadelphia.—ED.

